# Study of the Home-Auxiliary Robot Based on BCI

**DOI:** 10.3390/s18061779

**Published:** 2018-06-01

**Authors:** Fuwang Wang, Xiaolei Zhang, Rongrong Fu, Guangbin Sun

**Affiliations:** 1 School of Mechanic Engineering, Northeast Electric Power University, Jilin 132012, China; xiaolei_052@sina.com; 2 College of Electrical Engineering, Yanshan University, Qinhuangdao 066004, China; wangfuwangbaiyang@126.com; 3 Technology and Engineering Center for Space Utilization, Chinese Academy of Sciences, Beijing 100094, China; gbsun@csu.ac.cn

**Keywords:** home-auxiliary robot platform, physical disabilities, BCI, SL, autonomous return

## Abstract

A home-auxiliary robot platform is developed in the current study which could assist patients with physical disabilities and older persons with mobility impairments. The robot, mainly controlled by brain computer interface (BCI) technology, can not only perform actions in a person’s field of vision, but also work outside the field of vision. The wavelet decomposition (WD) is used in this study to extract the δ (0~4 Hz) and θ (4~8 Hz) sub-bands of subjects’ electroencephalogram (EEG) signals. The correlation between pairs of 14 EEG channels is determined with synchronization likelihood (SL), and the brain network structure is generated. Then, the motion characteristics are analyzed using the brain network parameters clustering coefficient (C) and global efficiency (G). Meanwhile, the eye movement characteristics in the F3 and F4 channels are identified. Finally, the motion characteristics identified by brain networks and eye movement characteristics can be used to control the home-auxiliary robot platform. The experimental result shows that the accuracy rate of left and right motion recognition using this method is more than 93%. Additionally, the similarity between that autonomous return path and the real path of the home-auxiliary robot reaches up to 0.89.

## 1. Introduction

As people age, their limb movement becomes more and more difficult, which brings inconvenience to their life. Additionally, some people can lose their normal neuromuscular pathway through which the brain exchanges information with the outside world. To solve this problem, brain computer interface (BCI) technology is developed by researches. BCI technology is a direct communication method established between the brain and a controlled device, which makes it possible to use the human brain to directly control external devices to assist humans in performing related operations [1,2,3,4]. The characteristics of human motion, which were collected in previous studies using models of the Event-Related Potential (ERP) [5,6,7,8] and the Steady-State Visual Evoked Potential (SSVEP) [9,10], are obvious. Additionally, these two models, which have strong anti-interference ability, are widely used in BCI technology research. A BCI system, which is highly recognized by users, should be non-invasive, safe, and practical to use. Many research teams have developed BCI systems which were used to control the navigation of humanoid robots [11,12,13,14,15,16,17,18,19], assistive exoskeletons [20], flying robots [21,22], robotic wheelchairs [21,23,24], and wheeled robots [25,26,27]. Moreover, the study of telepresence robots controlled by electroencephalogram (EEG) signals is an area of interest to researchers [25,28].

Research shows that the theory of modern complex networks has been used extensively to imitate human brain function [29]. Brain connectivity analysis has been proven to be a very effective and informative way to explore brain function and neural activity [30,31,32,33]. The theory of modern complex networks has been used to analyze human brain activities [34,35]. One study presented a small-world structure of the brain from the functional connectivity point of view, in which the functional relationship of the human brain is characterized by the structural properties of the network [36]. The two structural properties of the network, the clustering coefficient and global efficiency, are used to analyze the motion characteristics in this study. The electrooculogram (EOG), in experiments on BCI, was usually removed due to interference signal [37,38]. The authors applied BCI technology in combination with characteristics of EOG signals to control the auxiliary robot to complete the left-right movement. Additionally, the robot, which used the autonomous return mode to go back to the original starting point, could reduce the labor intensity of the users. This rendered it more convenient to use in the field of home application.

## 2. Experiment

### 2.1. Subjects

**Figure 1:**
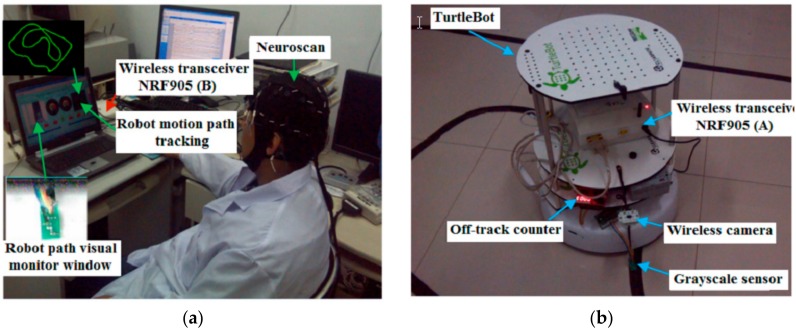
Experimental setup; (**a**) shows the experimental environment in which the subjects are located, (**b**) shows the TurtleBot and its running track.

A total of 10 healthy subjects (eight males and two females; aged 27 ± 1.3 (Standard Deviation) years), who were randomly selected from a group of volunteers, were chosen for this experiment. All of the subjects, who were required to meet the condition of no visual illness or history of neurological diseases, were asked to refrain from consuming any type of stimulus such as alcohol, tea, or coffee during the experiment. Figure 1 shows the experimental setup.

### 2.2. Experimental Process

The authors used Neuroscan as the EEG acquisition device, and its electrodes were attached to the scalp according to the international 10–20 system (30 channels = FP1, FP2, F7, F3, FZ, F4, F8, FT7, FC3, FCZ, FC4, FT8, T3, C3, CZ, C4, T4, TP7, CP3, CPZ, CP4, TP8, T5, P3, PZ, P4, T6, O1, OZ, and O2), which can detect the brain activity features reflected in frontal, central, and posterior regions of the human brain [39,40].

**Figure 2:**
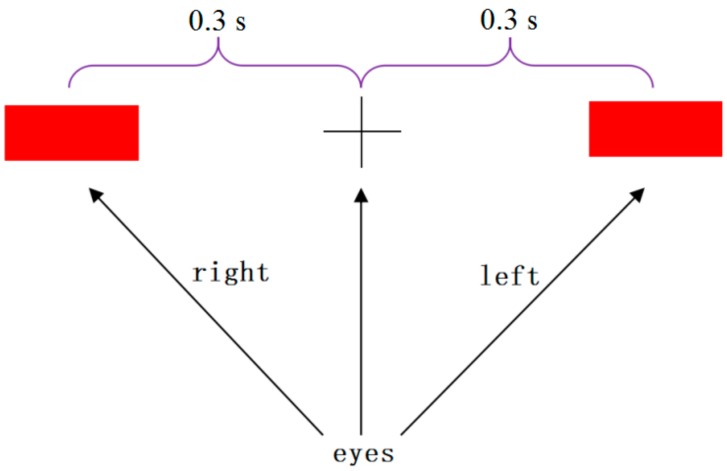
The model of visual evoked stimulation.

The subjects in this study were arranged in a room as shown in Figure 1a. The home-auxiliary robot (TurtleBot) was arranged in another room as shown in Figure 1b. Subjects controlled the robot to walk using the wireless video sensor and the wireless radio frequency communication device NRF905 (made in China by Zhejiang Hangzhou Yuze Electronic Company, Hangzhou, China). Two types of experimental actions were performed per subject to ensure that the robot’s grayscale sensor was not out of orbit. One action involved the subject looking at the cross mark (Figure 2), and then moving their eyes to the end of the left red rectangle. Then, the subject moved their eyes back to look at the cross mark, and then back to the robot’s path visual monitor window to monitor the robot walking. The eye movement process was completed within 0.3 s. The second action involved the subject looking at the cross mark (Figure 2), and then moving their eyes to the end of the right red rectangle, and then completing the process in the same way as described for the previous experimental action. The model of visual evoked stimulation is shown in Figure 2.

The robot received a steering command through the wireless device NRF905 (A) and turned 15 degrees each time. The robot would continue to travel at a speed of 0.05 m/s after the steering was completed until the next steering command was received. Each subject controlled the robot to walk along the track by using EEG and EOG signals, and then allowed the robot to autonomously return to its starting position along the original track. Additionally, the path the robot walked was recorded by a PC every time.

All subjects were informed about the research background and the study protocol. Moreover, all of them gave their written informed consent to be included in the study. The Ethics Committee at the Northeast Electric Power University Hospital endorsed the study protocol, according to the Code of Ethics of the World Medical Association (Declaration of Helsinki). Additionally, they were free to choose to participate in the experiment or resign.

### 2.3. Data Pre-Processing

The EEG signals were easily influenced by noise, so the raw EEG signals needed to be quieted first. The wavelet decomposition (WD), which was represented as a continuous time wavelet decomposition sampled at different frequencies at every level, specifically enabled the authors to discriminate between non-stationary signals with different frequency features [41]. Studies have shown that the WD is more efficient than other methods in the frequency domain [42]. The WD was defined as a continuous wavelet transform (*CWT*) and a discrete wavelet transform (DWT), and where the input signal was *X*(*t*), the *CWT* was defined as:

(1) $$CWT(a,b)=\int X(t)\psi_{a,b}^{*}(t)dt$$

where * denotes a complex conjugate, *a*∈*R*^+^ shows the scale parameter, *b*∈*R*^+^ shows the translation, and *ψ_a_*_,*b*_*(t)* was obtained by scaling the prototype wavelet *ψ(t)* at time *b* and scaling by *a* as follows:

(2) $$\psi_{a,b}^{}(t)=\frac{1}{\sqrt{a}}\psi (\frac{t-b}{a})$$

The orthogonal dyadic functions are often chosen as the mother wavelet in the WD, which is defined as:

(3) $$\psi_{j,k}(t)=2^{-j/2}\psi (2^{-j}t-k)$$

where {*ψ_j,k_*(*t*), *j*, *k*, ∈Z} for *L*^2^(*R*).

**Figure 3:**
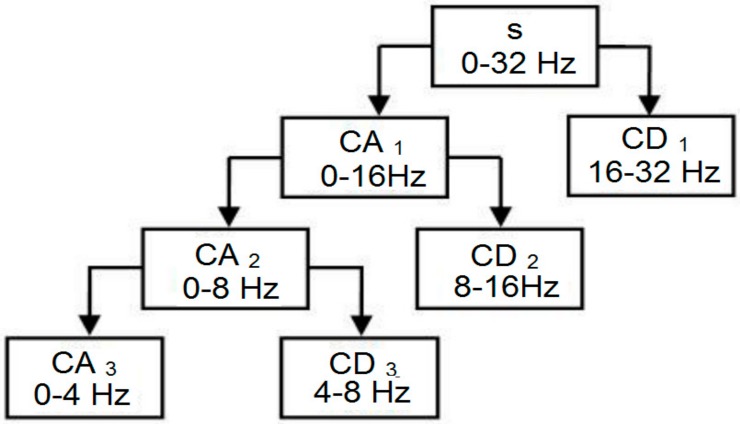
Sub-band coding algorithm.

The DWT method analyzed the signal at different frequency bands with different resolutions. The function, decomposing the signal into the different frequency bands, was completed using successive high-pass and low-pass filtering of the time domain signal. The original signal, which is shown as *x*[*n*], was first passed through a half-band high-pass filter *g*[*n*] and a low-pass filter *h*[*n*]. Then, half of the samples were eliminated according to Nyquist’s rule. This procedure can be represented as follows:

(4) $$Y_{high}[k]=\sum_{}^{}x[n]\times g[2k-n]$$

(5) $$Y_{low}[k]=\sum_{}^{}x[n]\times h[2k-n]$$

where *Y_low_* [*k*] and *Y_high_* [*k*] are the outputs of the low-pass and high-pass filters, respectively. Following this procedure, sub-sampling was performed twice. The sub-sampling process is presented in in Figure 3.

Butterworth high-pass and low-pass filters as well as the filtering of the EEG signals at 0.5 Hz and 32 Hz were used to remove artifacts and noisy signals. Then, EEG data were divided into low and high wavelet coefficients. Once again, these low and high wavelet coefficients were divided into their sub-high and sub-low wavelet coefficients. Resulting from the original EEG signal, the authors obtained *δ* (0~4 Hz) and *θ* (4–8 Hz) sub-bands.

## 3. Algorithm

### 3.1. Brain Network

Research has shown that a number of cortical and sub-cortical regions are activated in different brain regions when human beings process complex information [43]. Every region of the brain is taken as a node and the connections between brain regions are taken as edges. The brain connectivity analysis of the subjects was used to express the differences of nerve activity between the two brain hemispheres. The connection between pairs of EEG channels was determined with the synchronization likelihood (SL) and the calculation method described below.

Consider a time series given by *X_k__,i_* (K = 1, …, M; *i* = 1, …, *N*). With a given embedding dimension *m*, the series can be denoted as:

(6) $$X_{k,i}=(x_{k,i},x_{k,i+l},x_{k,i+2l},\ldots ,x_{k,i+(m-1)l})$$

The probability of the distance between pairs of embedded vectors less than *ε* is:

(7) $$P_{k,i}^{\varepsilon }=\frac{E_{i}}{2(\omega_{2}-\omega_{1})}\sum_{j=1}\theta (\varepsilon -|\overset{}{{\overset{N}{X}}_{k,i}-X_{k,j}}|)$$

where *ω*_1_ < |*j − i*| < *ω*_2_. The Euclidean distance is expressed as |•| and the Heaviside staircase function is expressed as *θ*. The *ω*_1_ and ω_2_ are two window variables, which meet the condition *ω*_1_ « *ω*_2_ « *N*.

Regarding each *k* and each *i*, we can determine a critical distance using *ε_k_*_,*i*_:

(8) $$P_{k,i}^{\varepsilon_{k,i}}=P_{ref}$$

in which *P_ref_* « 1. The number of channels, whose distance is less than the critical distance between vectors *X_k_,_i_* and *X_k_,_j_*, is expressed as:

(9) $$H_{i,j}=\sum_{K=1}^{M}\theta (\varepsilon_{k,i}-|\overset{}{{\overset{}{X}}_{k,i}-X_{k,j}}|)$$

in which *ω*_1_ < |*j − i*| < *ω*_2_. The SL algorithm, which defines each channel and each discrete time, is expressed as:

(10) $$S_{K,i,j}=\frac{H_{i,j}-1}{M-1}$$

where |*X_k_,_i_* − *X_k_,_j_*| < *ε_k,i_* and the average is calculated for all *j* values, which can be expressed as:

(11) $$S_{k,i}=\frac{I}{2(\omega_{2}-\omega_{1})}\sum_{j=1}^{N}S_{k,i,j}$$

in which *ω*_1_ < |*j − i*| < *ω*_2_.

The correlations between pairs of 14 channels (14 channels = F7, F3, F4, F8, FT7, FT8, C3, C4, TP7, TP8, P3, P4, O1, and O2) were calculated using Equation (11). Then, the brain networks were formed. The steps of the brain connectivity analysis are described as follows.

First, the data of a 14-channel EEG were collected. Then, the δ and θ sub-bands were extracted from the EEG signals. The adjacent matrix was computed for the sub-bands (δ and θ). Each matrix element corresponded to the SL between a pair of channels EEG signals. The entries on the main diagonal were equal to 1, because each EEG signal was perfectly correlated with itself. The matrix elements were symmetric relative to the main diagonal, that is, C*_ij_* = C*_ji_*. An edge was deemed to exist between *i* and *j* if their SL was greater than the fixed T; otherwise, no edge existed between *i* and *j*. Finally, the networks were formed using the adjacent matrix and a threshold value.

**Figure 4:**
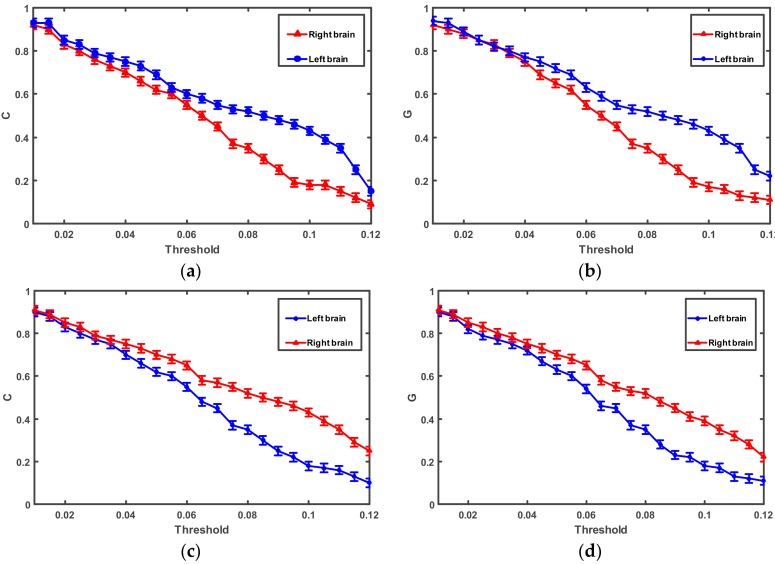
(**a**) Mean clustering coefficient (C) as a threshold when moved to the right; (**b**) mean global efficiency (G) as a threshold when moved to the right; (**c**) mean C as a threshold when moved to the left; (**d**) mean G as a threshold when moved to the left.

The SL values lie in the range Pref_T_ ≤ T ≤ 1, where the Pref_T_ is the minimum value which is close to 0, or close to 1 in the case of maximally synchronous signals. To compare the clustering coefficient (C) and global efficiency (G) of brain networks between the left and right brain hemispheres, networks were formed for the two brain hemispheres. The authors explored a whole range of the T values, 0.01 < T < 0.12, with increments of 0.005. Figure 4 shows the comparison of C and G at different brain hemispheres.

Over the whole range of threshold values (0.01–0.12), the significant difference of C can be found in Figure 4 for different brain hemispheres when T is in the range of 0.07 < T < 0.11. The significant difference of G can be found for different brain hemispheres when T is in the range of 0.08 < T < 0.11. The authors used the mean value of T (T = 0.092) for the correlation calculation so, the mean value of T (T = 0.092) was chosen as the fixed threshold. Using the fixed threshold, the network parameters C and G for all of the subjects’ brain networks were computed.

C and G were used to analyze the functional differences of the complex brain networks. These are explained in the following subsections.

#### 3.1.1. Clustering Coefficient

The connectivity degree of a node indicates the importance of that node in a network, which can be represented as the number of edges connected to that node. C can be expressed as the ratio of the number of existing edges to the number of maximum possible edges [44,45]. Its formula can be represented as:

(12) $$C_{i}=\frac{E_{i}}{D_{i}(D_{i}-1)/2}$$

in which *E_i_* is the number of existing edges between neighbors of the node *i* and *D_i_* is the degree of connectivity of that node. *D_i_*(*D_i_*–1)/2 is the number of maximum possible edges between neighbors of the node *i* [45].

#### 3.1.2. Global Efficiency

G can express the degree of integration of a network, which is associated with the speed at which the human brain processes information. The path length *L_i_*_,*j*_ between two nodes *i* and *j* is the minimum number of edges that are needed to connect. Additionally, the path length, which is the inverse ratio of the nodal efficiency, is mathematically defined as [44,45]:

(13) $$L_{i}=\frac{1}{N-1}\sum_{i\neq j\in G}L_{i,j}$$

where *L_i_*_,*j*_ is the minimum path length. The *N* is the number of nodes within a network. The average value of the nodal efficiencies of each node can be used to estimate the G. Thus, the global efficiency (G) of nodes can be defined by:

(14) $$G=E_{global}=\frac{1}{N(N-1)}\sum_{i\neq j\in G}\frac{1}{L_{i,j}}$$

Equation (14) can express that networks, which are characterized by a short minimum path length between any pair of regional nodes, have high global efficiency [46,47]. Combined with Equation (12), this leads to the fact that the bigger the values of C and G, the faster the information transmission speed of a node with others.

### 3.2. Motion Feature Recognition

**Figure 5:**
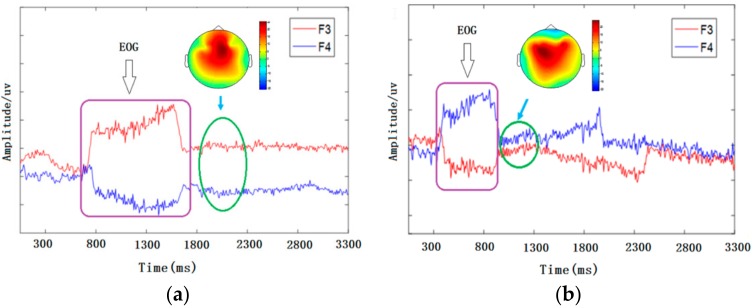
The difference regarding eye movement and brain topography when moved right (**a**) and left (**b**).

Obvious fluctuations occurred in the F3 and F4 channels when eyes move left or right, and the directions of fluctuations are opposite. The result is shown in Figure 5.

Following the fluctuations that occurred in the F3 and F4 channels, the brain topography showed that there was a significant difference between the left and right hemispheres, which indicated that there were differences in neural activities between the two hemispheres. Regarding brain topography, low activity is indicated by the blue-shaded areas, whereas high activity is indicated by the red-shaded areas. Figure 5a shows that the color of the right-brain region is darker than the color of the left-brain region, which means that the neural activities in the right-brain are more active than those in the left-brain when a person is moving to the left. Additionally, a different phenomenon appears, as shown in Figure 5b, when a person is moving to right.

A moving window with a width of 20 samples was established in the experiment, and the fluctuation characteristics of the eye movement signal were identified by using Equation (15).

(15) $$K_{i}=\frac{y(x_{i+20})-y(x_{i})}{20}$$

The K values of the F3 and F4 channels display opposite fluctuation changes when a person’s eyes move to the left or to the right. Concurrently, this meets the |K| > 1 condition. The authors used these features (G, C, and K) to judge the direction of movement for the robot.

**Figure 6:**
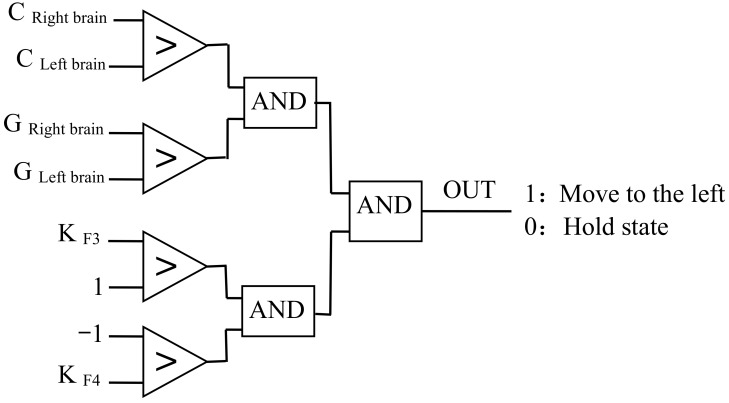
The logic of motion recognition.

The eye movement fluctuations in the time domain signal were identified using Equation (15), the parameter values for the left hemisphere and the right hemisphere were calculated. Then, the direction of the subject’s motion according to the K value and the brain network parameters’ value were judged. Taking the left movement as an example, the discrimination logic is shown in Figure 6.

### 3.3. Autonomous Return

**Figure 7:**
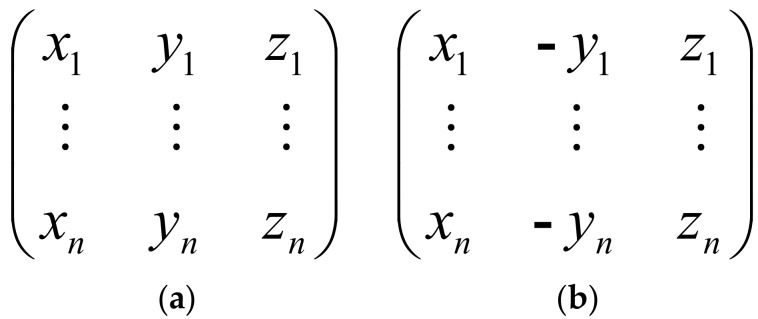
Robot motion track array. (**a**) Running track array; (**b**) return track array.

The TurtleBot walked along the track to the destination controlled by EEG and EOG signals. During the robot travel, the software recorded each direction conversion information as an array for the robot, which included the robot walking time, steering direction, and steering angle elements. The arrays are shown in Figure 7.

Figure 7a shows the running track array, in which *x* is the robot walking time, *y* is the steering direction (1: turn to the left and −1: turn to the right), and *z* is the steering angle. Subjects controlled the robot to walk along the track using their EEG and EOG signals, and the direction conversion information was recorded in the running track array. Subsequently, the return track array was transformed from the running track array using a simple mathematical algorithm. Finally, the TurtleBot autonomously returned to its starting position along the original track using the return track array.

## 4. Results

The characteristics of human motion feature signals were comprehensively identified by combining the characteristics of the human brain network with the characteristics of eye motion signals in motor imagery.

### 4.1. Motion Recognition

**Figure 8:**
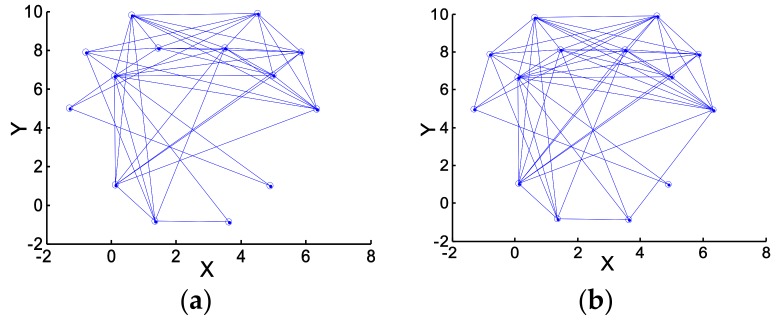
The difference of brain networks when moving right and left. (**a**) Move to the left; (**b**) move to the right.

The correlations between pairs of 14 EEG channels (14 channels = F7, F3, F4, F8, FT7, FT8, C3, C4, TP7, TP8, P3, P4, O1, and O2) were calculated using Equation (11), following which the brain networks were formed. Figure 8 shows the brain networks of one subject when he turned right and left in the experiment.

**Figure 9:**
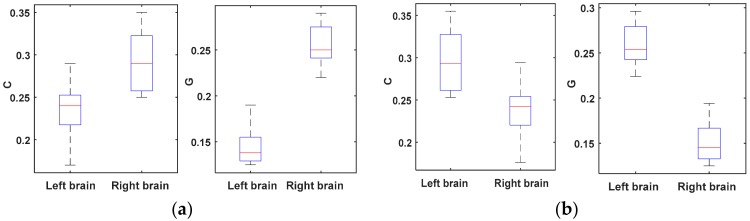
The difference of brain network parameters when a subject turns right or left. (**a**) Move to the left; (**b**) move to the right.

One clearly can see from Figure 8 that the connection density of the right-brain network is significantly higher than that of the left-brain network when the subject turned left. Quite the opposite, the connection density of the left-brain network is significantly higher than that of the right-brain network when the subject turned right. To quantify the density of a brain network, the parameters clustering coefficient (C) and global efficiency (G) were used to calculate and analyze network characteristics. Figure 9 shows the comparison of the brain network parameters C and G between a subject turning left and turning right.

Figure 9 shows that there are significant differences in C and G when subjects turn to the left and right (*P* < 0.05). Taking the left movement as an example, the connectivity density of the right hemisphere was higher than that of the left hemisphere when ERP was used to stimulate left motor imagery. The corresponding brain network parameter values (C and G) of the right hemisphere were larger than those of the left hemisphere at that time. This means that the neuron cluster in each brain region of the right hemisphere of the human brain cooperated to complete an equivalent action at that time, and the correlation between them was strong. Meanwhile, the neuron cluster in each brain region of the left hemisphere of the human brain did not need to cooperate to complete an equivalent action, thus the correlation between them was weak.

The authors analyzed motion recognition accuracy using EOG and EEG. Meanwhile, the accuracy was analyzed separately using the EOG and EEG signals. The accuracy comparison of motion direction recognition is shown in Table 1.

Table 1 shows that the accuracy rate of left and right motion recognition using EEG and EOG is more than 93%. This is compared with the identification methods using EOG and EEG separately, demonstrating that the recognition rate of the proposed method in this paper is higher.

### 4.2. Track Similarity

**Figure 10:**
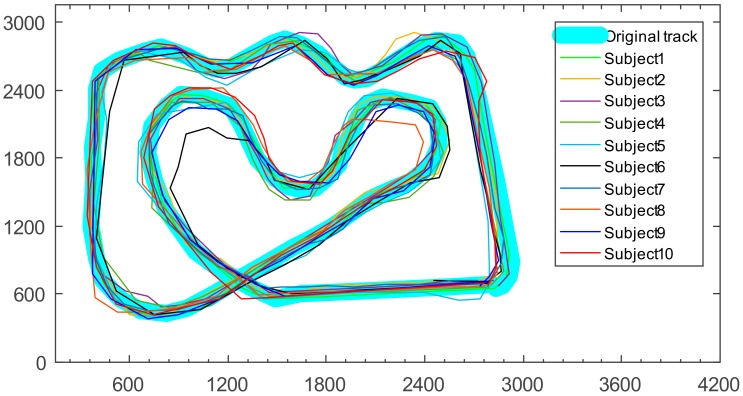
Track of autonomous return.

Subjects controlled the robot to walk along the track by using EEG and EOG signals, and then allowed the robot to autonomously return to its starting position. Additionally, the track of the robot’s autonomous return to its starting position was record by the PC, which is shown in Figure 10. The experiment was conducted eight times for each subject, and the track data of each subject were averaged.

The track similarity of the TurtleBot between the autonomous return track and the experimental original track reflects the accuracy of the robot motion controlled by EEG and EOG. To determine the similarity of the two types of track, the authors calculated the Pearson’s correlation coefficient between them. The similarity values calculated for the subjects are shown in Table 2.

Table 2 shows that the values of correlation coefficients between the autonomous return track and the experimental original track are greater than 0.50. This means that these variables have a strong correlation. Specifically, the correlation coefficients are greater than 0.85 for subjects after six tests. Thus, one can conclude that the method applied in this paper has high control precision after proper training for subjects, which makes it more convenient to use in the field of home application.

### 4.3. Training Effect

**Figure 11:**
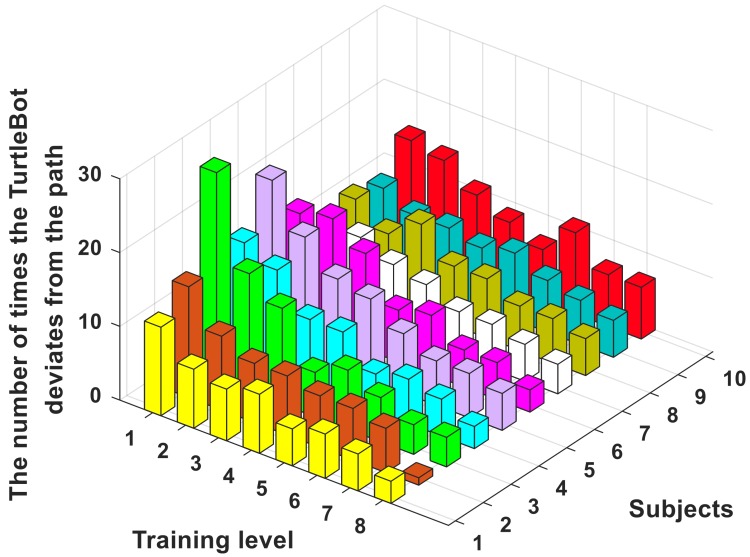
The number of times the TurtleBot deviates from the path.

Each subject was tested eight times. The number of times the robot deviated from the path was recorded by the off-track counter shown in Figure 1b. The results are shown in Figure 11.

Figure 11 shows that the number of times the TurtleBot deviated from the path decreases significantly as the amount of training increases, which means that the method using EEG and EOG to control the robot’s movement has high accuracy after proper training. Additionally, the autonomous return of the robot makes it more convenient to use in the field of home application.

## 5. Discussion

The authors used EEG and EOG to comprehensively identify and control the TurtleBot to walk along the track and return by using the autonomous return mode, which made it more convenient to use in the field of home application.

### 5.1. Previous Studies

Rinku Roy et al. used the Genetic Algorithm (GA) to identify left-right arm movement. They achieved a correct recognition rate of 75.77% [48]. CAS Filho et al. used the graph method to classify human hands signals, achieving a high recognition rate of up to 98% [49]. Research shows that the recognition of motor information in brain signals using the BCI is an effective method [50,51,52]. Although it is very accurate to identify motion characteristics using EEG, the authors cannot confirm that it also has an efficient motion recognition rate when the auxiliary robot is controlled in real time outside the field of vision.

### 5.2. Novel Findings of This Study

This study’s results show that the method that subjects used to control the robot to walk along the track by using the EEG and EOG had high precision of the controls and recognition speed. Additionally, the method can record the data of the TurtleBot walking track, which can make the robot autonomously return to its starting position, all of which makes it more convenient to use in the field of home application.

### 5.3. Limitations and Future Research Lines

The EEG acquisition equipment with high precision is expensive and inconvenient to wear, which is not conducive to the popularization and application of this technology. Additionally, this study is limited to using EEG, EOG, and related sensors to quickly control the TurtleBot’s path of movement and return. Future research works might develop portable equipment that can be conveniently worn and inexpensively fabricated. It could become a reality that an individual could control a manipulator to easily imitate their motion using motion imagination.

## 6. Conclusions

A home-auxiliary robot platform was developed which could facilitate patients with physical disabilities and older persons with mobility impairments. The authors applied BCI technology to practical operations. Combined with ERP vision evoked stimulation, the method, which applies portable EEG acquisition equipment, acquires EEG signals and extracts EEG motion characteristics in real time. The experimental result showed that the accuracy rate of left and right motion recognition using this method is more than 93%. Additionally, the similarity between the autonomous return track and the original track of the home-auxiliary robot reached up to 0.89. Thus, one can conclude that the method applied in this paper has high control precision after proper training for subjects, which makes it more convenient to use in the field of home application.

## Figures and Tables

**Table 1:** Accuracy comparison of motion direction recognition.

	EEG	EOG	Comprehensive Detection Using EEG and EOG
Subject 1	80%	90%	98%
Subject 2	85%	95%	100%
Subject 3	90%	85%	95%
Subject 4	85%	88%	99%
Subject 5	85%	90%	93%
Subject 6	80%	85%	97%
Subject 7	83%	94%	98%
Subject 8	79%	87%	96%
Subject 9	88%	91%	97%
Subject 10	77%	87%	94%

**Table 2:** The similarity between the autonomous return track and the experimental original track.

Number of Experiments	Subject 1	Subject 2	Subject 3	Subject 4	Subject 5	Subject 6	Subject 7	Subject 8	Subject 9	Subject 10
1	0.7832	0.7533	0.6523	0.7398	0.6813	0.7761	0.8237	0.7934	0.8021	0.7731
2	0.8567	0.7821	0.6822	0.7678	0.7165	0.7324	0.7895	0.8277	0.8314	0.7964
3	0.8927	0.8175	0.7127	0.8136	0.7837	0.7752	0.8109	0.7899	0.8237	0.8311
4	0.8465	0.8013	0.8397	0.8287	0.8019	0.8532	0.8369	0.8322	0.8417	0.8543
5	0.9235	0.8618	0.8156	0.9213	0.8515	0.8314	0.8562	0.8452	0.8253	0.8827
6	0.9011	0.8728	0.8993	0.8986	0.9089	0.9210	0.8613	0.8871	0.8657	0.8375
7	0.9207	0.8922	0.9204	0.9185	0.9102	0.9125	0.8943	0.8793	0.8955	0.8815
8	0.9345	0.9688	0.9158	0.9281	0.9054	0.9227	0.9328	0.9127	0.9003	0.8966
